# Transcriptomic analysis between Normal and high-intake feeding geese provides insight into adipose deposition and susceptibility to fatty liver in migratory birds

**DOI:** 10.1186/s12864-019-5765-3

**Published:** 2019-05-14

**Authors:** Guosong Wang, Long Jin, Yan Li, Qianzi Tang, Silu Hu, Hengyong Xu, Clare A. Gill, Mingzhou Li, Jiwen Wang

**Affiliations:** 1 Farm Animal Genetic Resources Exploration and Innovation Key Laboratory of Sichuan Province, Sichuan Agricultural University, Chengdu, Sichuan 611130 People’s Republic of China; 2 Department of Animal Science, Texas A&M University, College Station, TX 77843 USA

**Keywords:** Adipogenesis, Goose, Transcriptome

## Abstract

**Background:**

Dysregulation of adipogenesis causes metabolic diseases, like obesity and fatty liver. Migratory birds such as geese have a high tolerance of massive energy intake and exhibit little pathological development. Domesticated goose breeds, derivatives of the wild greyleg goose (*Anser anser*) or swan goose (*Anser cygnoides*), have high tolerance of energy intake resembling their ancestor species. Thus, goose is potentially a model species to study mechanisms associated with adipogenesis.

**Results:**

Phenotypically, goose liver exhibited higher fat accumulation than adipose tissues during fattening (liver increased by 3.35 fold than 1.65 fold in adipose), showing a priority of fat accumulation in liver. We found the number of differentially expressed genes in liver (13.97%) was nearly twice the number of that in adipose (6.60%). These differentially expressed genes in liver function in several important lipid metabolism pathways, immune response, regulation of cancer, while in adipose, terms closely related to protein binding, gluconeogenesis were enriched. Typically, genes like *MDH2* and *SCD,* which have key roles in glycolysis and fatty acids metabolism, had higher fold change in liver than in adipose tissues. Three hundred two differentially expressed long noncoding RNAs involved in regulation of metabolism in liver were also identified. For example, lncRNA *XLOC_292762*, which was 5.7 kb downstream of *FERMT2,* a gene involved phosphatidylinositol-3,4,5-trisphosphate binding, was significantly down-regulated after the high-intake feeding period. Further investigation of documented obesity-related orthologous genes in goose suggested that understanding the evolutionary split from mammals in adipogenesis will make goose fatty liver a better resource for future research.

**Conclusions:**

Our research reveals that goose uses liver as the major tissue to regulate a distinct lipid synthesis and degradation flux and the dynamic expression network analyses showed numerous layers of positive responses to both massive energy intake and possible pathological development. Our results offer insights into goose adipogenesis and provide a new perspective for research in human metabolic dysregulation.

**Electronic supplementary material:**

The online version of this article (10.1186/s12864-019-5765-3) contains supplementary material, which is available to authorized users.

## Background

The balance of energy storage and energy expenditure is critical for normal adipose deposition and lipid metabolism. Adipose tissue has been recognized as a major endocrine organ and acts as the host for adipogenesis in mammals [1]. Excessive energy intake results in an increase in the volume and weight of adipocytes and causes dysregulation of lipid metabolism in the body [2]. Such dysregulation is reflected by variable lipid deposition in different adipose tissues, and it is usually associated with abnormal liver lipid accumulation, which can lead to steatosis and obesity [3]. In contrast to mammals, migratory birds show distinct lipid deposition patterns and use liver instead of adipose tissue as the main organ for lipid metabolism [4–6].

As a typical species domesticated from a migratory bird, goose (*Anser anser or Anser cygnoides*) has a completely different mechanism of lipid deposition from mammals and from some terrestrial poultry, such as chicken [7, 8]. A goose has the capability of depositing excess lipid in its liver. Research has demonstrated that the formation of goose fatty liver shares similar phenotypic changes with human non-alcoholic fatty liver, but differs in pathological development because goose fatty liver only shows a low level of inflammation and other immune responses [7, 9, 10]. This distinct difference in the phenotype indicates that goose fatty liver might become a resource for better understanding lipid deposition in birds and for human fatty liver research. Previous research on the synthesis and delivery of fatty acids focused on goose liver without considering adipose tissues [7, 11, 12]. To explore the dynamic genetic pattern behind the regulation of lipid deposition in goose, we have built a weight-gain model to investigate the mechanism. We collected and performed RNA-seq on liver, subcutaneous adipose, and abdominal adipose to better understand the expression network. Through comprehensive analysis, we showed that goose regulates lipid metabolism differently to mammals and that liver plays the most important role in this metabolic process. We envision that goose is a model for understanding lipid metabolism.

## Results

### Phenotypic changes of liver and adipose tissues after high-intake feeding

**Fig. 1:**
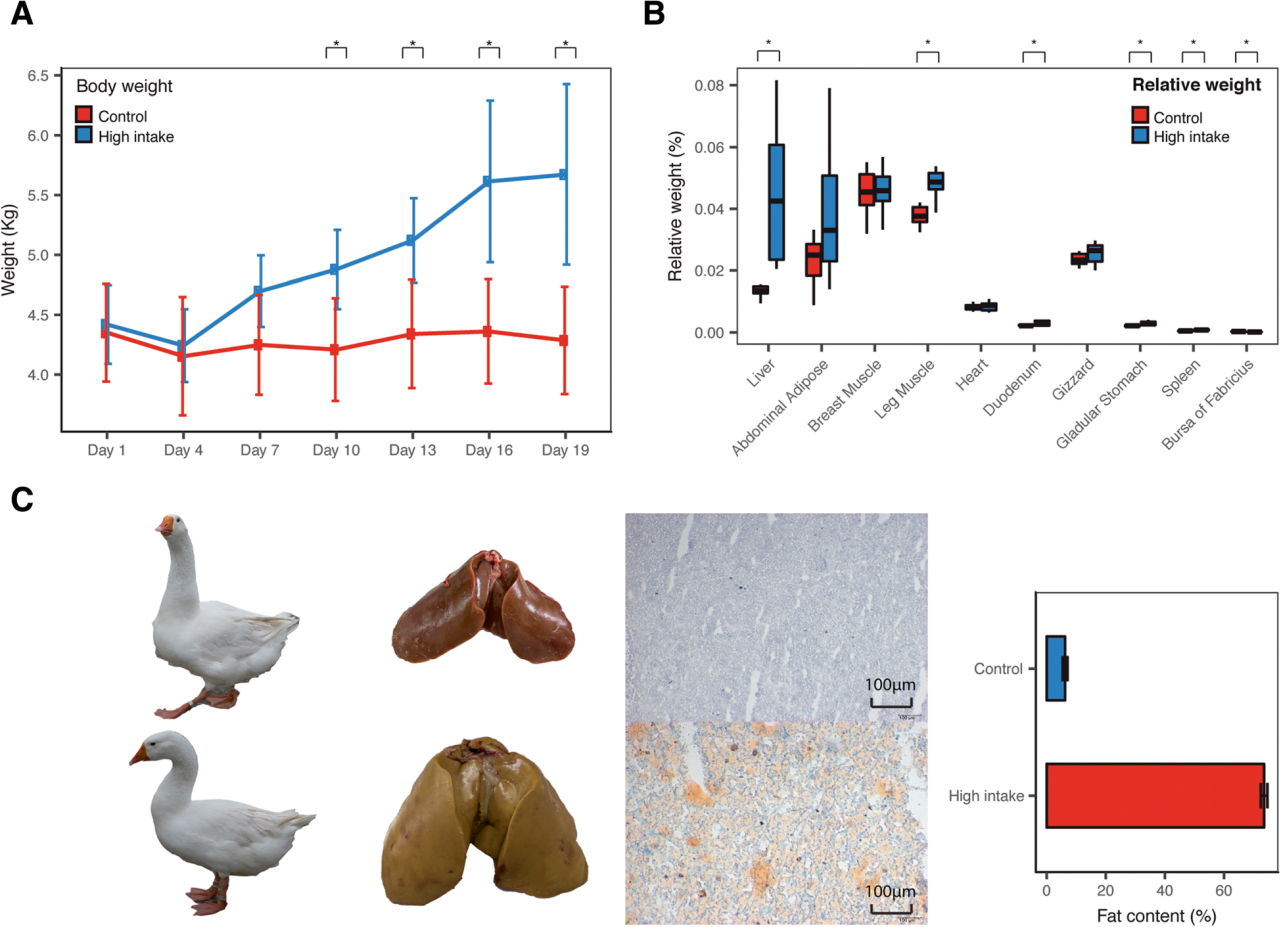
Phenotypic differences between normal and high-intake fed geese. **a** Sequential changes in weight. **b** Absolute weight and relative weight of tissues. Relative weight = absolute weight/body weight. **c** Enlarged photos of liver, corresponding frozen sectioned red oil staining and Soxhlet extraction of lipid content of livers. Photos of geese and livers were taken from the geese used in this study

Body weight increased 32.3% by the end of the fattening process (*p*-value = 8.30*10^− 4^, fold-change = 1.32) and the high intake group was significantly heavier beginning at day 10 of the fattening period (Fig. 1a, Additional file 7). Tissues related to lipid metabolism were heavier after fattening, with liver increasing the most in relative weight by 3.35 fold (*p*-value = 0.0011, Fig. 1b, Additional file 8), comparing to abdominal adipose increased by 1.65 fold. Moreover, after we assessed the lipid content of liver, we found it increased drastically from 6.22 ± 0.83% to 73.56 ± 1.14% (Fig. 1c), which largely contributed to the increased weight of the fatty liver. Red oil staining followed by integrated optical density (IOD) also confirmed substantial lipid deposition in liver (Fig. 1c, Additional file 1).

### Transcriptomic difference of liver and adipose tissues after high-intake feeding

To investigate dynamic expression changes induced by high-intake feeding, we generated an average of ~ 11.50 Gb high-quality RNA-seq data. We identified an average of 77.44% protein-coding genes with FPKM ≥0.1 and 1702 putative lncRNAs (Most lncRNAs were sense intergenic lncRNAs (44.6%), followed by divergent lncRNAs, and other 3 categories, Additional file 2). These lncRNAs showed similar expression characteristics with other research [13].

There were substantial differences between tissues in both mRNA profiles and lncRNA profiles (weighted average proportion variance = 0.47 and 0.55, respectively), followed by either interaction between treatment and tissue (weighted average proportion variance = 0.16 in mRNA profiles) or treatment (weighted average proportion variance = 0.16 in lncRNA profiles), indicating the major driver of the differences in expression is tissue, and the treatment effect on lncRNA expression patterns is bigger than mRNA expression patterns (Additional file 3).

**Fig. 2:**
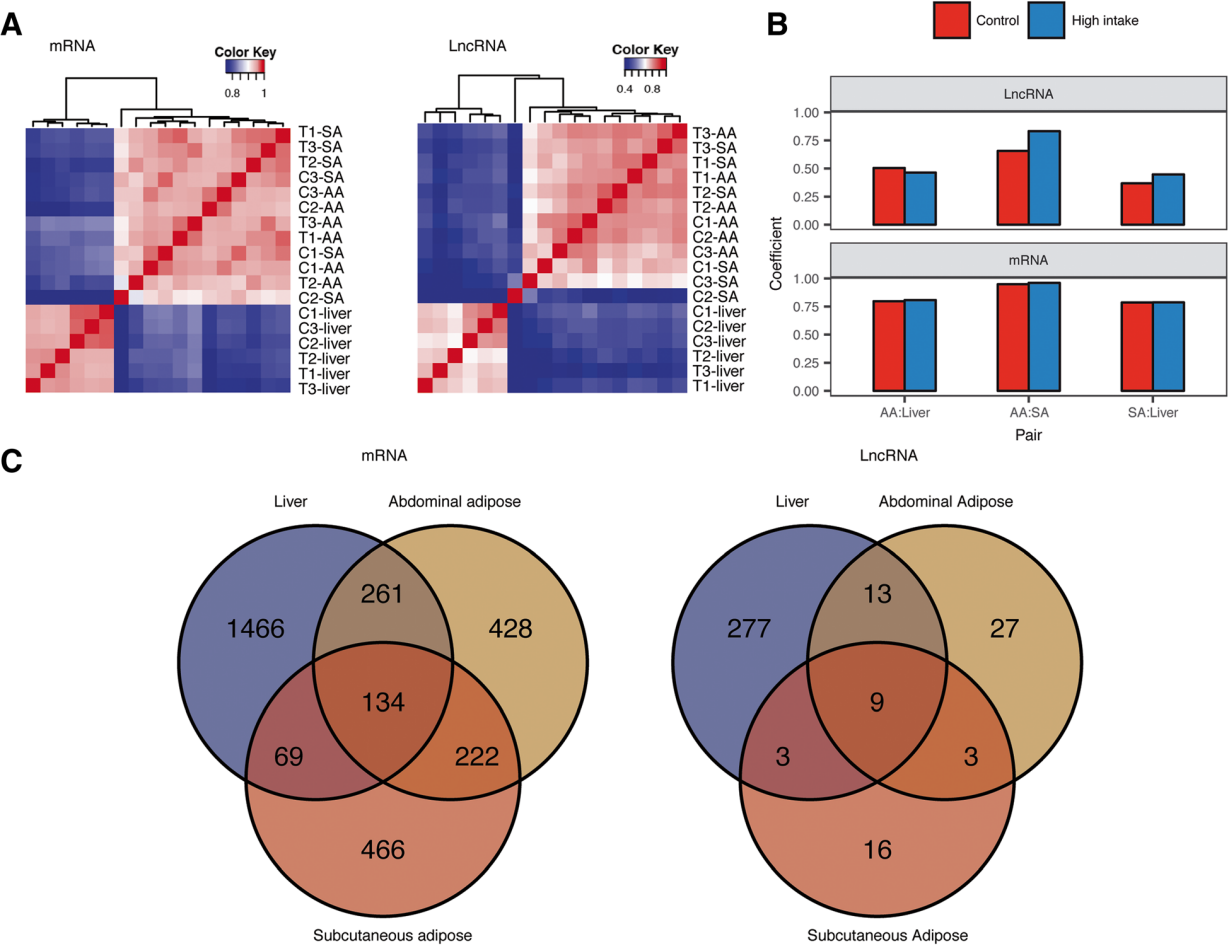
Transcriptomic changes for each tissue and each group. **a** Pearson’s correlation matrix for mRNA profiles and lncRNA profiles. **b** Within-group correlation of each pair of tissues. **c** Venn diagram of number of differentially expressed genes between normal fed geese and high-intake fed geese. AA – abdominal adipose, SA – subcutaneous adipose. T – high-intake group, C – control group

Unsupervised clustering also recapitulated the distinct expression pattern between liver and adipose tissues (Fig. 2a). Tissue-dominated clustering patterns and the distinct between-group liver clustering patterns revealed the overwhelming differences between tissues, and liver was more affected by treatment. Within-group correlation between tissues also confirmed the lower correlation between adipose and liver than between two adipose tissues (Fig. 2b). Control-group sample C2-SA showed lower correlation to adipose tissues in general, we hypothesize that this sample is partially contaminated with tissues such as skin.

### Protein coding genes involved in dynamic lipid metabolism

We found more than 6000 DEGs between liver and adipose tissues (under both normal and high-intake feeding conditions), compared to only ~ 400 DEGs between the two adipose tissues. We identified 1930 DEGs in liver (13.97% of 13,815 expressing genes with FPKM > 0.1), compared to 1045 (6.60% of 15,829 genes with FPKM > 0.1) and 891 DEGs (4.73% of 18,839 genes with FPKM > 0.1) in abdominal adipose and subcutaneous adipose after high-intake feeding, respectively (Fig. 2c). The detection of more than twice the number of DEGs in liver compared to adipose, and the large number of liver-specific DEGs supports the hypothesis that liver has a role in lipid metabolism during high-intake feeding.

**Fig. 3:**
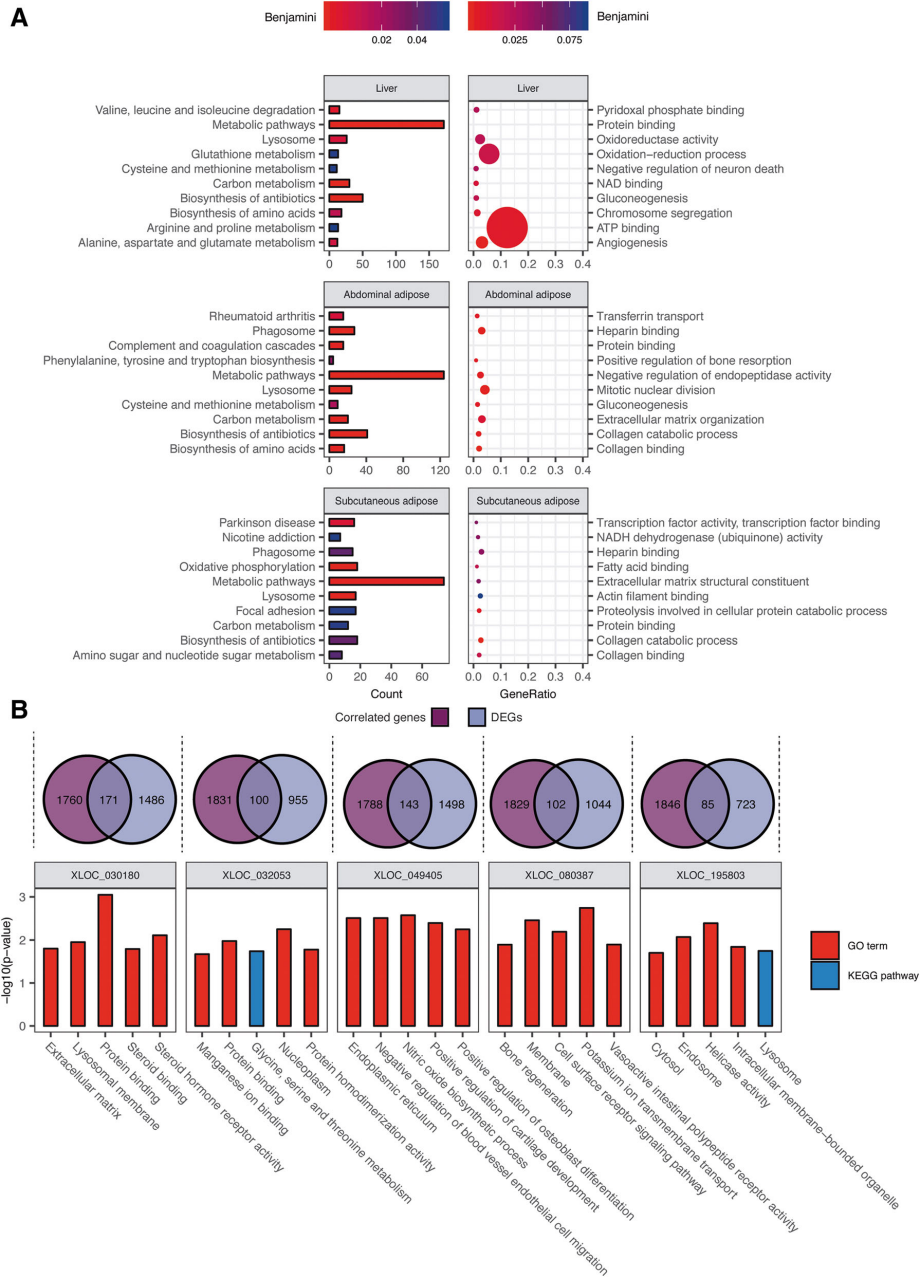
Enrichment of differentially expressed genes and lncRNA-correlated genes. **a** Top 10 GO/KEGG pathways of differentially expressed genes in liver, abdominal adipose and subcutaneous adipose. Left – enrichment of KEGG pathways, right – enrichment of GO terms. **b** Exhibition of the enrichment results of 5 lncRNAs correlated with differentially expressed genes (DEGs). Correlated genes were defined as genes associated with lncRNAs that have correlation coefficients over 0.80 and *p*-value < 0.05

As expected from the changes observed in phenotype, DEGs found in liver were significantly involved in metabolic pathways such as amino acids metabolism, carbon metabolism, and immune response. These genes tended to be functional in ATP binding, protein binding, oxidation-reduction process, and gluconeogenesis (Fig. 3a). Similar enrichment of DEGs found in abdominal adipose and subcutaneous adipose was observed and most of the enriched pathways and GO terms were related to metabolism as expected. The changes in metabolic processes were related to up-regulation of expression in the liver upon excess energy intake (Additional file 4). We also noticed that down-regulated DEGs were more involved in immune response, especially cancer-related pathways (Additional file 4). The association between expression changes and little or no pathological development in goose should be further investigated.

We found expression of 14 out of 20 previously identified goose mitochondria and important nuclear mitochondria-related genes [14]. The mitochondrial genes did not show a substantial response to high-intake feeding and most did not exhibit significant changes in expression in the 3 tissues we examined (Additional file 5). Gene enrichment analysis showed that nuclear genes related to the mitochondrial functions oxidation-reduction (adjusted *p*-value = 3.96*10^− 4^) and mitochondrial matrix (adjusted *p*-value = 8.54*10^− 10^) were significantly up-regulated (Additional file 6). Genes such as malate dehydrogenase 2 (*MDH2*) was up-regulated after high-intake feeding with liver showed the biggest change (fold-change = 2.01, 0.92 and 0.94 in liver, abdominal adipose and subcutaneous adipose, respectively). The increased intensity of mitochondrial metabolism is suggestive of elevated energy production and consumption, expected from the phenotypic changes and treatment.

### Long noncoding RNAs are related to fatty liver formation

**Fig. 4:**
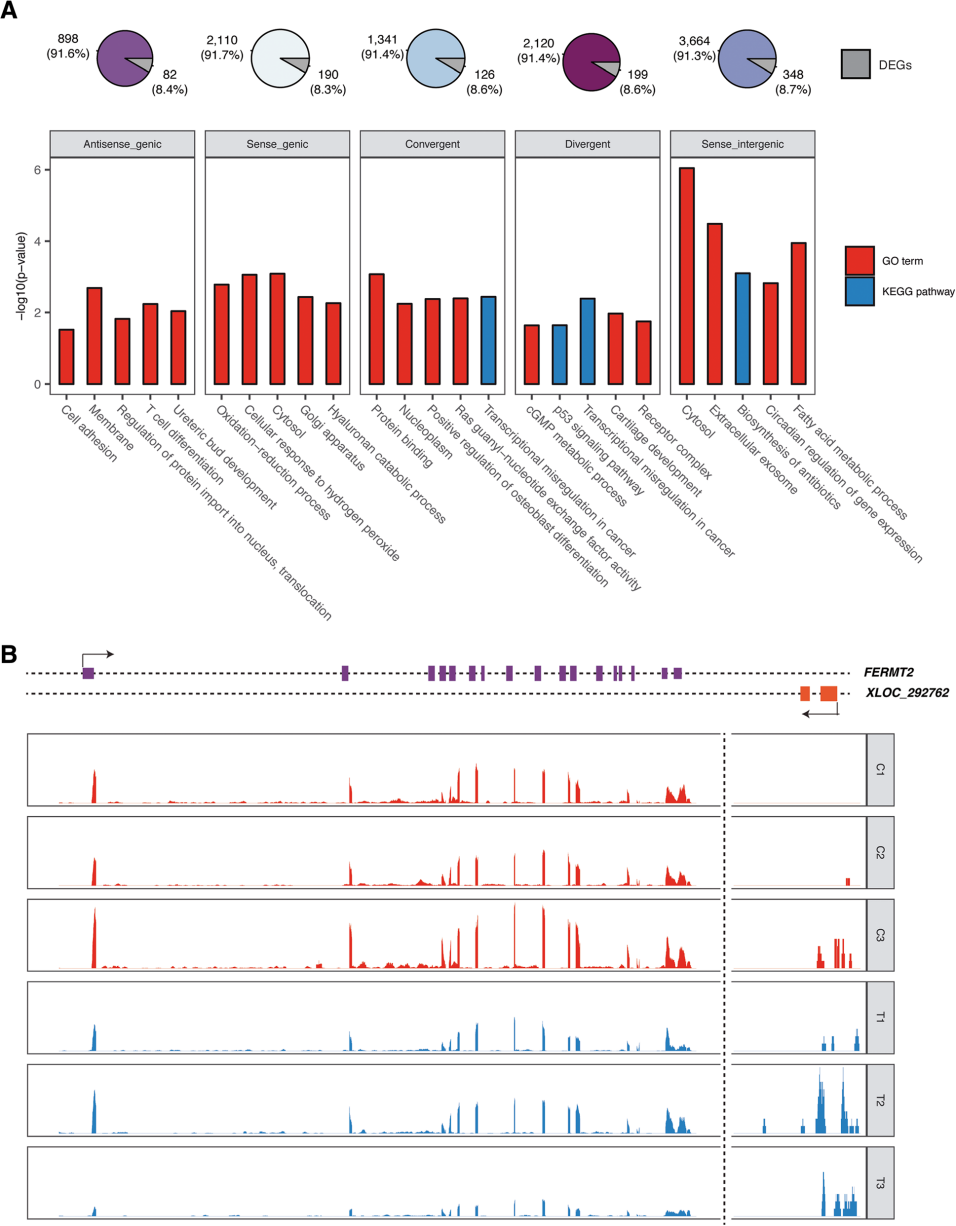
Prediction of lncRNA functions. **a** Enrichment of highly correlated mRNAs of different lncRNA types. LncRNAs were divided into 5 panels based on their type and were designated with a distinct color in the pie chart. Pie charts show the percentage of differentially expressed genes (DEGs) among correlated genes. **b** Exhibition of possible cis-acting lncRNA *XLOC_292762* which locates at 5700 bp downstream. The expression scale of *XLOC_292762* is different to FERMT2

Differences between liver and adipose tissues were also reflected by lncRNAs. Similar to protein coding genes, more differentially expressed (DE) lncRNAs were identified in liver (302 DE lncRNAs, 19.24% of 1570 lncRNAs with FPKM > 0.1) than adipose tissues (52 for abdominal adipose, 3.13% of 1662 lncRNAs with FPKM > 0.1 and 41 for subcutaneous adipose, 2.43% of 1689 lncRNAs with FPKM > 0.1), and the majority of these DE lncRNAs were specific to liver (Fig. 2c). To determine the role of lncRNAs in the formation of fatty liver, DEGs that were highly correlated to specific lncRNAs with the highest fold change after the high-intake feeding period showed a strong relationship to metabolism. Enriched terms included amino acids metabolism, protein binding, and endoplasmic reticulum, which was found as a usual response to high-intake feeding, suggesting lncRNAs may act as possible trans-acting regulators of protein coding genes (Fig. 3b). By narrowing down to different categories of lncRNAs, strong enrichment for metabolism and immune functions was also observed within each type (Fig. 4a). Among protein coding genes spatially associated with each type of lncRNA, an average of 8.5% of them were differentially expressed. Genes involved in fatty acid metabolism, cGMP metabolic processes, and oxidation-reduction were significantly enriched. Among protein coding genes located within 10 kb of the lncRNA, we found the genes *ZFAT*, *GJD2*, *HOXA10*, and *B3GALT2* (Additional file 9) that are related to metabolism, degradation of endoplasmic reticulum protein and transcription factors [15–17]. These nearby lncRNA-mRNA pairs are suggestive of cis-acting regulation of lipid metabolism during high-intake feeding. For example, *FERMT2,* which is involved phosphatidylinositol-3,4,5-trisphosphate binding, was possibly down-regulated by a downstream (− 5.7 Kb) intergenic antisense (convergent) lncRNA *XLOC_292762* that was up-regulated after the high-intake feeding period (Fig. 4b). Antisense lncRNAs have been identified functional in mammalian gene network. For example, they can regulate the expression of mRNA by forming an RNA-RNA duplex at the 5′ end of the mRNA which is important to 5′ end-dependent degradation pathways. The further exploration of antisense lncRNAs and other types of lncRNAs could provide more details about the regulatory functions of them.

## Discussion

The accumulation of lipid, mostly triacylglycerol (TAG), within hepatocytes is the key prerequisite for the development of non-alcoholic fatty liver disease (NAFLD) in humans. Serum non-esterified fatty acids (NEFA) are the most dominant source for lipid accumulation in liver [3]. The major sources contributing to the level of serum NEFA include NEFA derived from fatty acid flux in adipose tissues and from chylomicrons through lipoprotein spillover [18]. Fatty acid flux in adipose tissues uses the de novo lipogenesis pathway to synthesize fatty acids from 2-carbon precursors made from dietary glucose. Research has shown non-alcoholic fatty liver disease patients have elevated masses of visceral and subcutaneous adipose tissues. Current therapeutic strategies for NAFLD patients are focusing on the reduction of adipose tissue fatty acid flux [19], indicating the important role of adipose tissues in the regulation of lipid metabolism.

Among goose adipose tissues and liver, our results showed that goose liver was the only lipogenic tissue with significantly increased weight (Fig. 1b). However, another study using Tianfu geese showed a significant increase in abdominal adipose weight [20]. Other research has shown breed differences in capability to deposit fat [21]. In our study, we only found an increasing trend for abdominal adipose. In earlier work, aside from weight change, the percentage of lipid content in adipose tissue did not significantly change, while the percentage of lipid content in liver increased significantly [20]. This is consistent with our results and in contrast to fat content accumulation in mammalian adipose, which is the prioritized tissue for lipid deposition. In fact, the obesity in human was widely considered as a metabolic syndrome in adipose tissues [22], suggesting the important role of adipose tissues in mammals. These results indicated that lipid deposition in goose under high-intake feeding is different from mammals and this distinct difference in lipid deposition in liver and adipose tissues under high-intake feeding, compared to normal intake, implies there may be an intrinsic difference in transcriptional regulation. This adaptation may be related to long-distance migration, because poultry species like duck have a similar ability, whereas terrestrial poultry like chicken do not deposit lipid in similar way [7]. Interestingly, leg muscle also showed a significant increase in weight. We hypothesize this is an adaptation to the increased weight during the feeding process and an increase in the amount of intramuscular fat [23, 24]. Further experiments need to be done to determine whether it is an increase in muscle cell volume or an increase in the amount of intramuscular fat.

By investigating the expression patterns of protein-coding genes, the phenotypic difference was also reflected by the transcriptome-wide patterns. The clear segregation of liver expression profiles in protein coding genes and the lack of a dominant pattern of expression among adipose profiles showed that there was distinct difference between goose livers but not adipose tissues after normal feeding and high-intake feeding (Fig. 2a). The higher average correlation coefficients between the two adipose tissues also showed the high similarity between geese from the two feeding groups (Fig. 2b). Similar results have been observed in chickens that showed larger changes in liver than adipose tissues [25].

However, what we found by clustering the transcriptome-wide expression patterns does not necessarily reveal which genes are the drivers of the responses to feeding treatment. In order to accommodate more energy, it is reasonable to expect an increase in metabolism with high-intake feeding. Indeed, through our differential expression analysis, we revealed that genes related to metabolism were upregulated in liver and adipose tissues under this feeding treatment. For example, glycolysis, the first major metabolic pathway that breaks down glucose, was intensely upregulated after high-intake feeding. Phophoglucomutase-1 that controls the irreversible step of glycolysis was up-regulated [26]. Another important aspect about the formation of goose fatty liver is the balance between the storage and secretion of newly synthesized endogenous lipids and exogenous lipids in the plasma [7]. Key genes such as *FADS1* and *APOB* in this process were regulated, leading to lipid deposition in liver. We found the elevated level of stearoyl-CoA desaturase (*SCD*), an essential enzyme that transforms saturated fatty acids into unsaturated fatty acids [10], that was expressed nearly 3-fold higher in liver for geese fed a high-intake diet. This gene was also expressed 1.7-fold higher in abdominal adipose for the same diet, but not significantly differentially expressed in subcutaneous adipose. In the goose reference genome sequence [7] there were more *SCD* gene copies than chicken and expression of SCD was significantly increased after overfeeding. Moreover, we found *FADS* family and *DGAT2*, which both are involved in balancing lipid storage and degradation, were upregulated. This result demonstrates there is similarity between goose and mammals for lipid flux in adipose tissue, whereby the adipose tissue is contributing to the serum NEFA pool even though it may not be the major source of NEFA.

During the desaturation process mediated by *SCD*, two-carbon acetyl-CoA enters the citric acid cycle to generate ATP. The last few reactions of the citric acid cycle take place in mitochondria, instead of in the cytoplasm, and these steps are the final steps for beta-oxidation of fatty acids as well. Even though our results showed that genes from the mitochondrial genome were not differentially expressed, nuclear mitochondria-related genes were upregulated. These genes include *MDH2*, which encodes mitochondria-located malate dehydrogenase 2 and plays a central role in the malate-aspartate shuttle [27]. Our results are consistent with previous research who has identified five nuclear mitochondria-related genes were up-regulated in goose fatty liver [28], and again, the differences in expression levels of these genes in liver, abdominal adipose tissue, and subcutaneous adipose tissue support our observation that liver played the key role in the regulation process.

Usually, uncontrolled NAFLD will turn into nonalcoholic steatohepatitis, which is a more severe disease with liver cell inflammation and cell damage [29]. Nonalcoholic steatohepatitis is also a precursor for liver cancer. In our results, we found an interesting down-regulation of some important cancer pathways such as the PI3K-Akt and cGMP-PKG signaling pathways. These pathways are largely involved in processes like tumorigenesis [30, 31]. Our results showed the key genes of these pathways such as *PI3K* and *PKG* were down-regulated. Though it is too early to conclude the meaning of the down-regulation of these pathways, we believe our results provide evidence that goose develops little pathological development in fatty liver.

In addition to regulation by protein-coding genes, long noncoding RNAs have been recently identified in many species and function in many regulatory processes [32]. Typically, they are transcripts over 200 bp and are typically expressed at lower levels than protein-genes in cells [33]. they have been demonstrated to be functional in many biological processes [13, 32–34] who tend to be involved in many processes as cis- or trans-acting regulators [13]. To thoroughly understand the transcriptomic changes, we identified lncRNAs in goose for the first time and showed their possible roles in lipid metabolism. Expression patterns of lncRNAs were similar to protein coding genes in liver and the expression patterns of lncRNAs remained unclustered in adipose tissues. Our functional prediction for lncRNAs revealed the high possibility that they are actively involved in lipid metabolism and other processes in response to high-intake feeding. LncRNAs show high variation between species, which makes them hard to annotate based on sequence. Current computational prediction of the function of lncRNAs is based on correlated expression and their genomic location relative to protein coding genes. Although further functional validation is needed, our research has provided some evidence for the role of lncRNAs in goose.

**Fig. 5:**
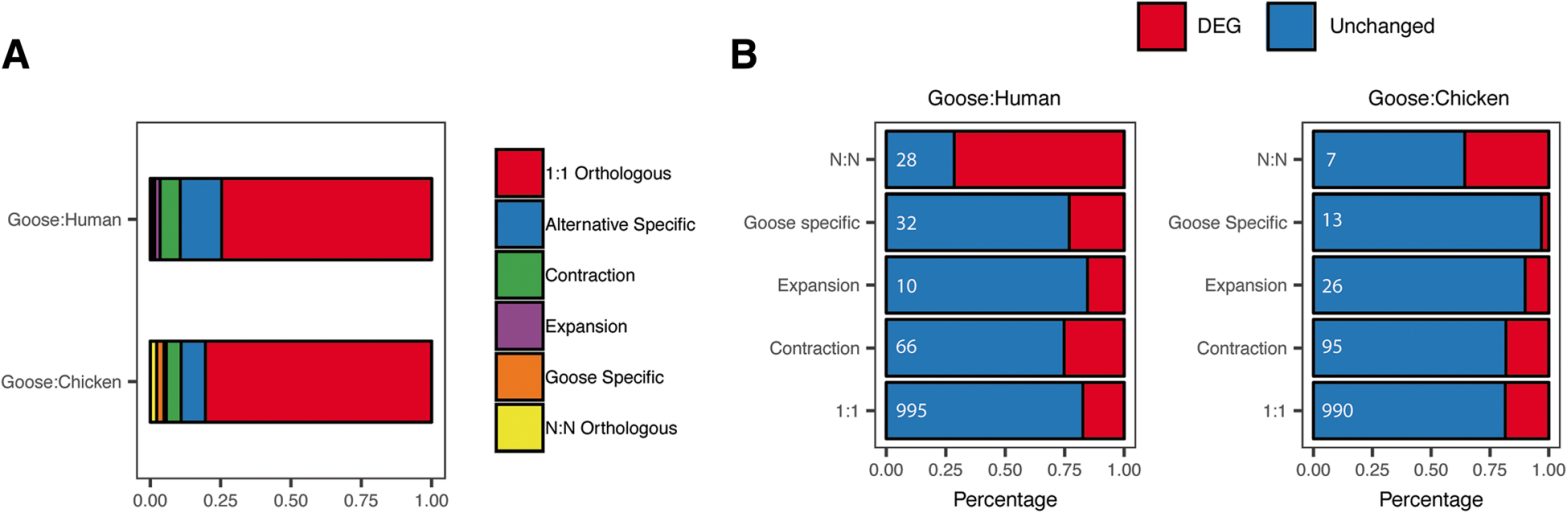
Orthologous relationship of 1519 mammal obesity genes. **a** Orthologous relationship of obesity genes between goose and chicken or goose and human. **b** Proportion of DEGs in each relationship category. Numbers marked on the bar show the total number of gene families in that category. Most of these genes shared a 1:1 orthologous relationship to human (74.66%) and more genes were 1:1 orthologous to chicken genes (80.53%). Some gene families showed either contraction (7.16 and 5.34% to human and chicken, respectively) or expansion (1.96 and 8.08% to human and chicken, respectively). Differentially expressed genes identified in liver also showed strong enrichment among these obesity genes (Chi-square *p*-value = 4.42*10^− 4^). Most DEGs shared a 1:1 orthologous relationship with human (or chicken) and accounted for 18% of all goose-human 1:1 obesity genes (or 18.49% of goose-chicken 1:1 obesity genes)

Recently, non-alcoholic fatty liver has been strongly associated with obesity in humans [35]. More than 1500 genes are strongly associated with obesity, in general, in mammals [36]. Among the more than 190 GO terms that have undergone rapid or slow evolution between goose and terrestrial birds based on previous research, GO terms including lipid binding, carbohydrate metabolic process and phosphatidylinositol phospholipase C activity were identified [7]. These results suggested that obesity-related genes may also be under selection to adapt to massive energy intake. There was a strong orthologous relationship between these obesity genes from mammals and goose genes (Fig. 5a), 1190 goose genes out of the 1518 mammalian genes identified to be orthologous to each other, the majority of orthologous genes shared a 1:1 orthologous relationship to human (74.66%). We think this suggests that genes related to obesity showed more sequence-level variants rather than change in copy number. Additionally, obesity-related genes had a significantly higher proportion of DEGs (Chi-square *p*-value = 4.42*10^− 4^), suggesting a possible functional regulation of obesity as well [36]. Most DEGs shared a 1:1 orthologous relationship with human (or chicken) and accounted for 18% of all goose-human 1:1 obesity genes (or 18.49% of goose-chicken 1:1 obesity genes) (Fig. 5b).

Given that we found ~ 80% of obesity genes were 1:1 orthologs in chickens and humans, and the fact that domesticated goose does not develop pathological hepatic steatosis upon high-intake feeding, we believe fully understanding the genetic mechanism behind the formation of goose fatty liver will help to uncouple the effects of obesity and human non-alcoholic fatty liver.

## Conclusions

In this study, we found that the goose uses liver as the major tissue to regulate a distinct lipid synthesis and degradation flux. To process extra energy, the regulation of gene expression is stronger in goose liver than in adipose tissues. This is different from the expression profiles in mammals. We demonstrated by dynamic expression network analyses that there are numerous layers of positive responses to both massive energy intake and possible pathological development. The ability to deposit large amounts of fatty acids in liver instead of adipose tissues, and the upregulation of many metabolic genes need further analysis to reveal the genetic mechanism behind. Our results offer insights into goose adipogenesis and provide a new perspective for research in human metabolic dysregulation.

## Methods

### Animals and their treatment

All animal handling procedures were approved by Sichuan Agricultural University Animal Welfare Committee. Twenty healthy 126-day-old males of the Tianfu meat geese breed were obtained from Sichuan Agricultural University Waterfowls Breeding Farm. Tianfu is a lean Chinese commercial breed that is an *A. anser* x *A. cygnoides* hybrid. The breed is a composite of 87.5% Landes (*A. anser)* and 12.5% of Sichuan White (*A. cygnoides)*. The population used in this study is closed and has been under selection for over 10 generations. We randomly assigned geese to two groups (*N* = 10). All geese had free access to water at all times. All geese were given the same diet (14.29 MJ/Kg metabolizable energy, 8.09% crude protein and 0.14% methionine) but the two groups differed in daily feeding times. The control group had access to diet freely the whole day and on average consumed 325 g (energy level 4.64 MJ/Kg) of feed a day. The feeding procedure for the high-intake feeding group was similar to what is used in the industry to produce foie gras. The birds were artificially force fed two times a day for days 1–3, 3 times a day for days 4–6, 4 times a day for days 7–17, and 3 times on day 18. The meals on day 1 were 130 g and were increased by 5 g every day. By the end of the feeding period the difference in energy intake between the two group was 72.81 MJ for each goose. On day 18, the day before the birds were sacrificed, all geese were deprived of feed overnight and they were sacrificed the following morning at 145 days of age through cervical dislocation. Body weight was recorded every 3 days for each goose.

### Sample collection and total RNA-seq

We collected liver, abdominal adipose, subcutaneous adipose, heart, breast muscle, leg muscle, duodenum, gizzard, glandular stomach, spleen and bursa of fabricius from each goose. Tissue weights were collected and tissue samples were put into liquid nitrogen immediately after being extracted from body.

For total RNA sequencing, we randomly selected 3 individuals as biological replicates from both the normal and the high-intake feeding group. Total RNA from liver, abdominal adipose, and subcutaneous adipose for each individual (a total of 18 samples) were extracted with the using RNeasy Mini Kit (QIAGEN, Germany) following the manufacturer’s instructions. RNA integrity was checked by Agilent Bioanalyzer 2100 (Agilent Technologies, CA, USA). Samples with average RIN value = 7.59 (from 7 to 8.6) were then sent to Novogene (Tianjin, China) to generate paired-end libraries. All libraries were sequenced by Illumina Hiseq X 10 following Illumina’s protocols by Novogene with a read length of 150 bp. Standard quality control and filtering of low quality reads was performed by Novogene and clean reads were provided for further analysis.

### Transcriptome alignment and assembly

Clean reads were mapped against goose reference genome that includes the mitochondrial genome (AnsCyg_PRJNA183603_v1.0) using Bowtie2 [37] and the spliced reads aligner, Tophat2 [38] with default arguments. Tophat2 utilizes splicing information from the reference annotation file (GTF file) to guide the mapping of RNA-seq reads. BAM files for each library were then assembled by Cufflinks (V2.1.1) [39] using the *–g* argument, which invokes assembly of transcripts based on the reference annotations, de novo assembly of transcripts using a probabilistic model, and quantifies the expression of assembled transcripts at the same time. Relative expression was reported as fragments per kilobase of transcripts per million mapped reads (FPKM). Cufflinks generated assembled transcripts in GTF file format for all individuals and these were later merged by tissue using Cuffmerge. For all annotated genes, Pearson’s correlation coefficients of expression were calculated across the 18 samples. Principle variance components analysis (PVCA) was carried out using the R package pvca (https://www.bioconductor.org/packages/release/bioc/html/pvca.html).

### Identification of long noncoding RNAs

We used a homology-based method and constructed a personalized pipeline to identify putative long noncoding RNAs (lncRNAs). First, transcripts in the 6 merged GTF files (3 tissues × 2 treatments) were compared to the reference annotations using Cuffcompare and those transcripts that were in the reference GTF file or had a large overlap with a known transcript were removed. Removed transcripts were marked as “c” or “=” by the custom Python script. By default, transcripts shorter than 200 bp were not be retained. Protein-coding score were then calculated for remaining transcripts using CPC [40]. The protein-coding score is derived from comparison of a support vector machine learning classifier against a selected protein database. We calculated coding potential against UniRef 90 and transcripts with scores larger than 1 were filtered because of their high coding potential. Surviving transcripts were then scanned for potential of coding small peptides or functional protein domains using Infernal cmscan [41], which makes prediction using a Hidden Markov Model against a selected protein database. Here we used also UniRef 90 and again we filtered transcripts with high coding potential. Survived transcripts were then compared by blastx [42] to the NCBI NR database and UniRef 90. We filtered any transcripts that only had one exon and retained the rest of them as putative lncRNAs. As we did for protein coding genes, we used Cufflinks to quantify the expression of the lncRNAs, and we performed principle variance components analysis and hierarchical clustering analysis.

### Identification of differentially expressed genes and functional enrichment analysis

Cuffdiff was used to identify differentially expressed genes (DEGs) between tissues within the same treatment and between treatments for each tissue. Cuffdiff performs a pair-wise test for each pair of genes and corrects for multi-test bias at the same time using an FDR correction. Enrichment analysis between treatments was performed using the DAVID online enrichment tool (https://david.ncifcrf.gov) [43]. We first extracted the HGNC gene symbol for DEGs. We then converted HGNC symbols to human ENSEMBL IDs and applied these in DAVID. We performed the enrichment analysis for 3 gene ontology (GO) terms (biology process, molecular function, cellular component) and KEGG pathways. We separated enriched GO terms and KEGG pathways, and based on an adjusted Benjamini *p*-value, we selected the top 10 enriched GO terms and KEGG pathways for each tissue. We also separated DEGs into up-regulated and down-regulated DEGs and repeated the enrichment analysis.

### Analysis of putative lncRNAs

We calculated the average length and number of exons of lncRNAs. Briefly, we categorized putative lncRNAs into sense intergenic lncRNAs, sense genic lncRNAs, convergent lncRNAs, divergent lncRNAs, and antisense intergenic lncRNAs [44]. As a prediction of possible functions of lncRNAs, we annotated the lncRNAs based on their proximity to nearby protein-coding genes. Specifically, lncRNAs that were within 10 kb upstream or downstream of a protein-coding gene were marked as possible cis-acting lncRNAs. Correlation analysis for expression of all genes and lncRNAs was performed by using the R package Hmisc (https://cran.r-project.org/web/packages/Hmisc/). LncRNA and mRNA pairs with correlation coefficients > 0.80 and *p* < 0.05 were identified as correlated pairs. Customized python scripts were used to extract pairs that were both correlated and close to each other.

### Identification of orthologous gene families

We downloaded the coding sequences (CDS) and peptide sequence of goose, human, and chicken from NCBI. When there was more than one alternative transcript of a gene, we only kept the transcript with the longest CDS and we filtered any transcripts that coded for fewer than 50 amino acids. Protein sequences from the final set of transcripts were subjected to BLAST [42] to obtain the similarity between sequences (e-value was set to 1*10^− 5^). Hcluster_sg (https://github.com/douglasgscofield/hcluster) was used to cluster the results and T-Coffee [45] to rank the clusters. Finally, a customized script was used to categorize gene families (such as single-copy gene families, multi-copy gene families, and species-specific gene families).

## Additional files

Additional file 1: **Figure S1.** Oil red staining of adipose tissues and integrated optical density of three tissues. (PDF 2908 kb)

Additional file 2: **Figure S2.** Comparison of characteristics of protein coding genes and lncRNAs and classification of lncRNAs. (PDF 163 kb)

Additional file 3: **Figure S3.** Principle components variance analysis of mRNA and lncRNA profiles. (PDF 509 kb)

Additional file 4: **Figure S4.** Pathway enrichment of up-regulated DEGs and down-regulated DEGs. (PDF 359 kb)

Additional file 5: **Figure S5.** Expression of mitochondria genes and important nuclear mitochondria-related genes. Asterisk mark indicates significantly differentially expressed between normal and high-dietary fed geese. (PDF 133 kb)

Additional file 6: **Figure S6.** GO enrichment of up-regulated/down-regulated DEGs. (PDF 806 kb)

Additional file 7: **Table S1.** Paired t-test of body weights between normal and high dietary group during the fattening process. (PDF 63 kb)

Additional file 8: **Table S2.** Paired t-test of relative tissue weight between normal and high dietary group. Relative weight = absolute weight/body weight. (PDF 70 kb)

Additional file 9: **Table S3.** Top 10 highly correlated mRNA-lncRNA pairs within 10Kb of each other. (PDF 77 kb)

## Abbreviations

Akt: The serine-threonine protein kinase
ATP: Adenosine triphosphate
B3GALT2: Beta-1,3-galactosyltransferase 2
CDS: The coding sequence
cGMP: cyclic guanosine monophosphate
CPC: Coding potential calculator
DE: Differentially expressed
DEGs: Differentially expressed genes
FDR: False discovery rate
FERMT2: Fermitin family member 2
FPKM: Fragments per kilobase of exon per million reads
GJD2: Gap junction protein delta 2
GO: Gene ontology
GTF: Gene transfer format
HOXA10: Homeobox A10
kb: Kilobase
KEGG: Kyoto Encyclopedia of Genes and Genomes
lncRNA: long noncoding RNA
MDH2: Malate dehydrogenase 2
NAFLD: Non-alcoholic fatty liver disease
NEFA: Non-esterified fatty acids
PI3K: Phosphoinositide-3-Kinase
PKG: Protein kinase cGMP-dependent 1
PVCA: Principle variance components analysis
RIN: RNA integrity
SCD: Stearoyl-CoA desaturase
TAG: Triacylglycerol
ZFAT: Zinc finger and AT-hook domain containing
