# Supplementary figures and images for: Transcriptomic analysis between Normal and high-intake feeding geese provides insight into adipose deposition and susceptibility to fatty liver in migratory birds
Source: BMC Genomics. 2019 May 14;20:372. doi: 10.1186/s12864-019-5765-3 (PMC6518675; doi:10.1186/s12864-019-5765-3)

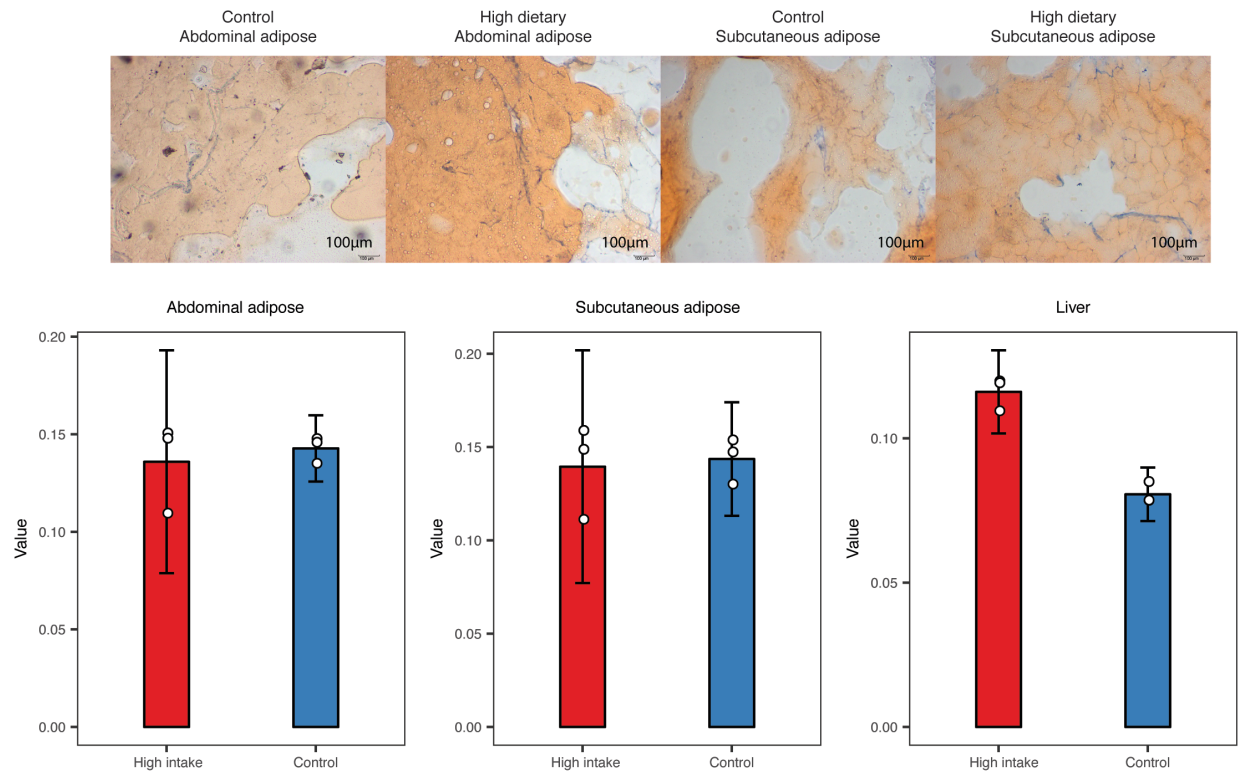

**Figure S1.** Oil red staining of adipose tissues and integrated optical density of three tissues.

Supplement: Supplementary file 1 — Figure S1. Oil red staining of adipose tissues and integrated optical density of three tissues. (PDF 2908 kb) [file 12864_2019_5765_MOESM1_ESM.pdf]

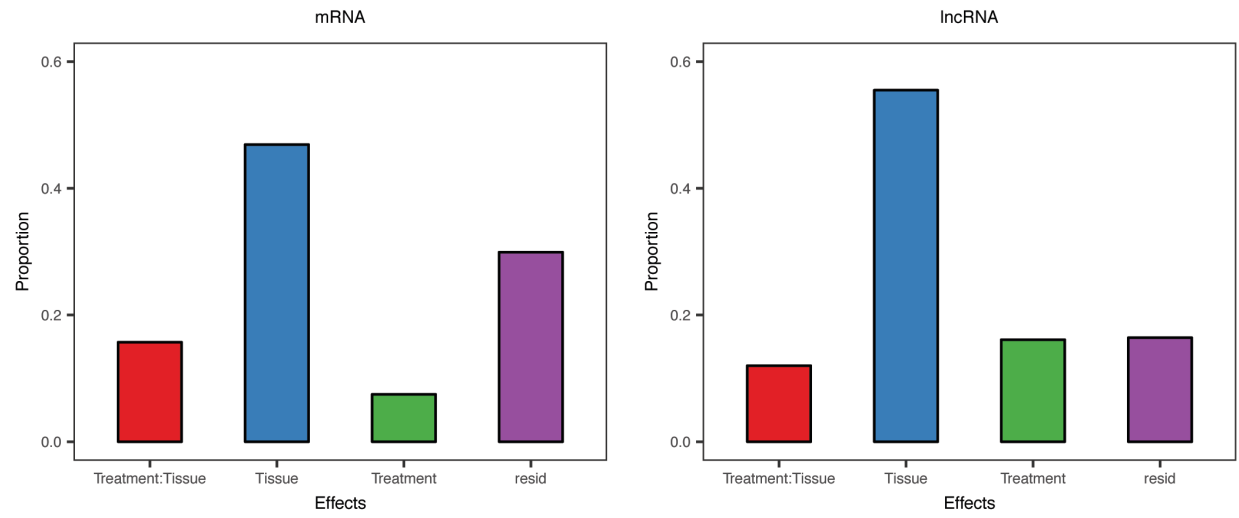

**Figure S3.** Principle components variance analysis of mRNA and lncRNA profiles.

Supplement: Supplementary file 3 — Figure S3. Principle components variance analysis of mRNA and lncRNA profiles. (PDF 509 kb) [file 12864_2019_5765_MOESM3_ESM.pdf]

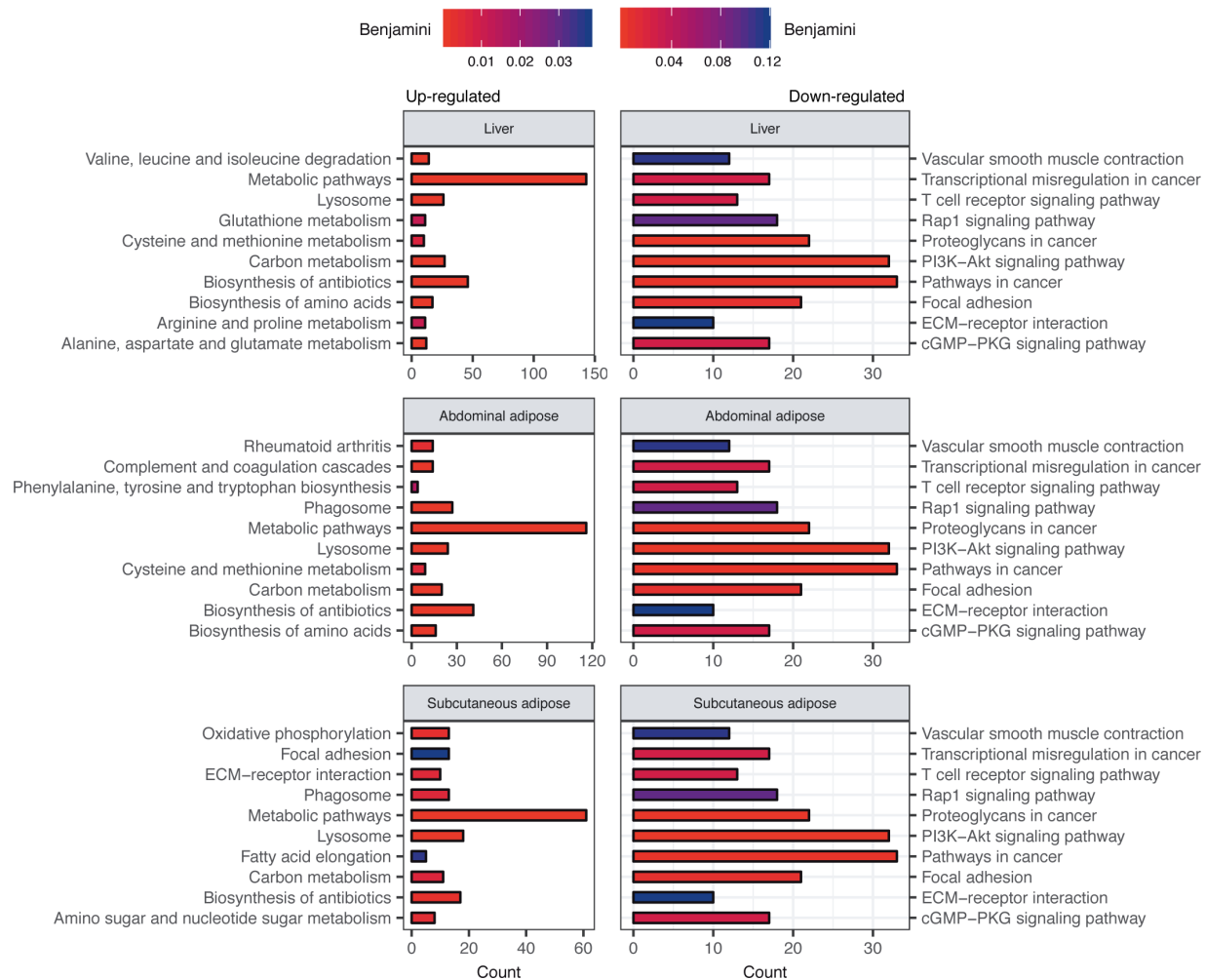

**Figure S4.** Pathway enrichment of up-regulated DEGs and down-regulated DEGs.

Supplement: Supplementary file 4 — Figure S4. Pathway enrichment of up-regulated DEGs and down-regulated DEGs. (PDF 359 kb) [file 12864_2019_5765_MOESM4_ESM.pdf]

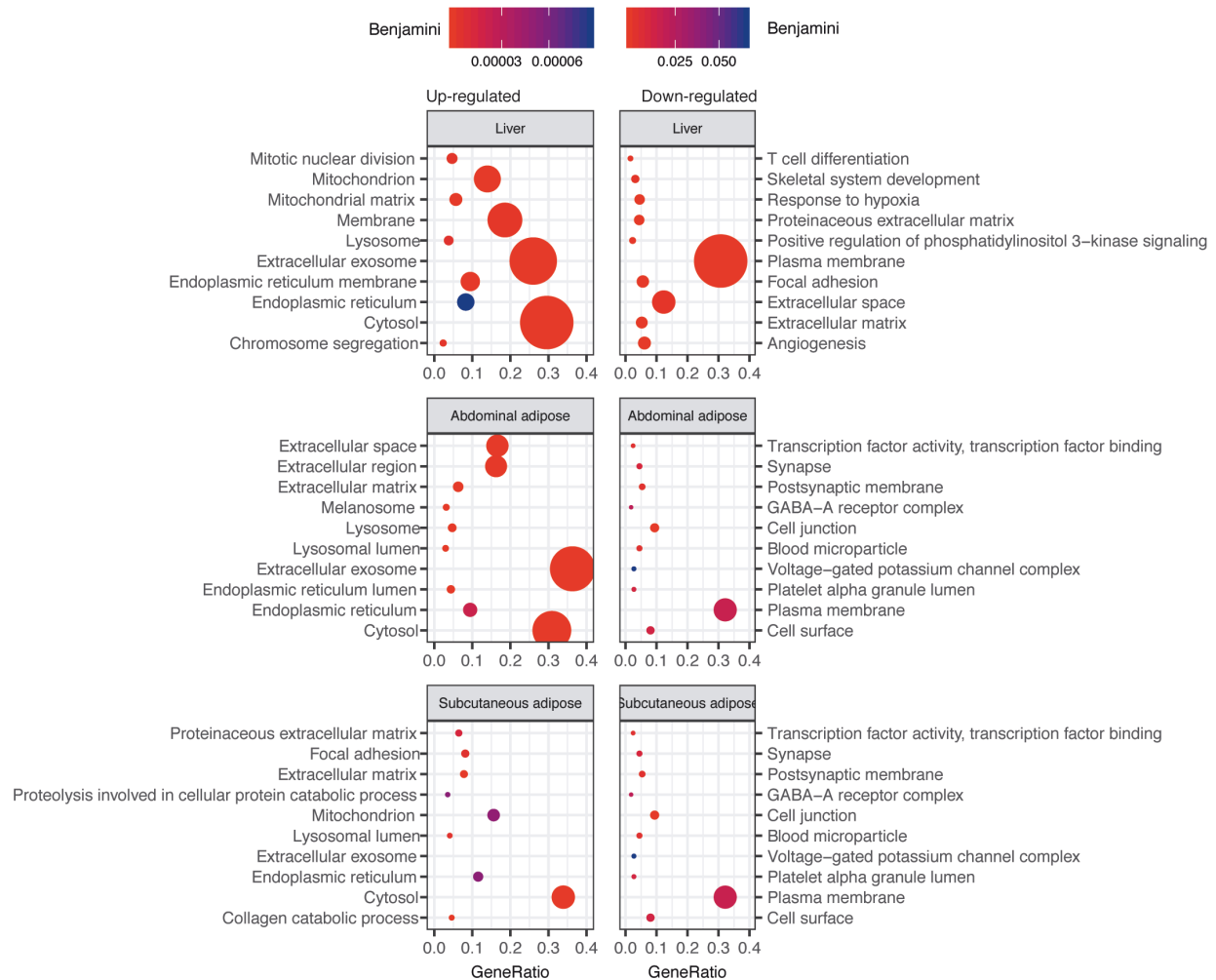

**Figure S6.** GO enrichment of up-regulated/down-regulated DEGs.

Supplement: Supplementary file 6 — Figure S6. GO enrichment of up-regulated/down-regulated DEGs. (PDF 806 kb) [file 12864_2019_5765_MOESM6_ESM.pdf]
