# Supplementary material for: Transcriptomic analysis between Normal and high-intake feeding geese provides insight into adipose deposition and susceptibility to fatty liver in migratory birds
Source: BMC Genomics. 2019 May 14;20:372. doi: 10.1186/s12864-019-5765-3 (PMC6518675; doi:10.1186/s12864-019-5765-3)
Supplement: Supplementary file 2 — Figure S2. Comparison of characteristics of protein coding genes and lncRNAs and classification of lncRNAs. (PDF 163 kb) [file 12864_2019_5765_MOESM2_ESM.pdf]

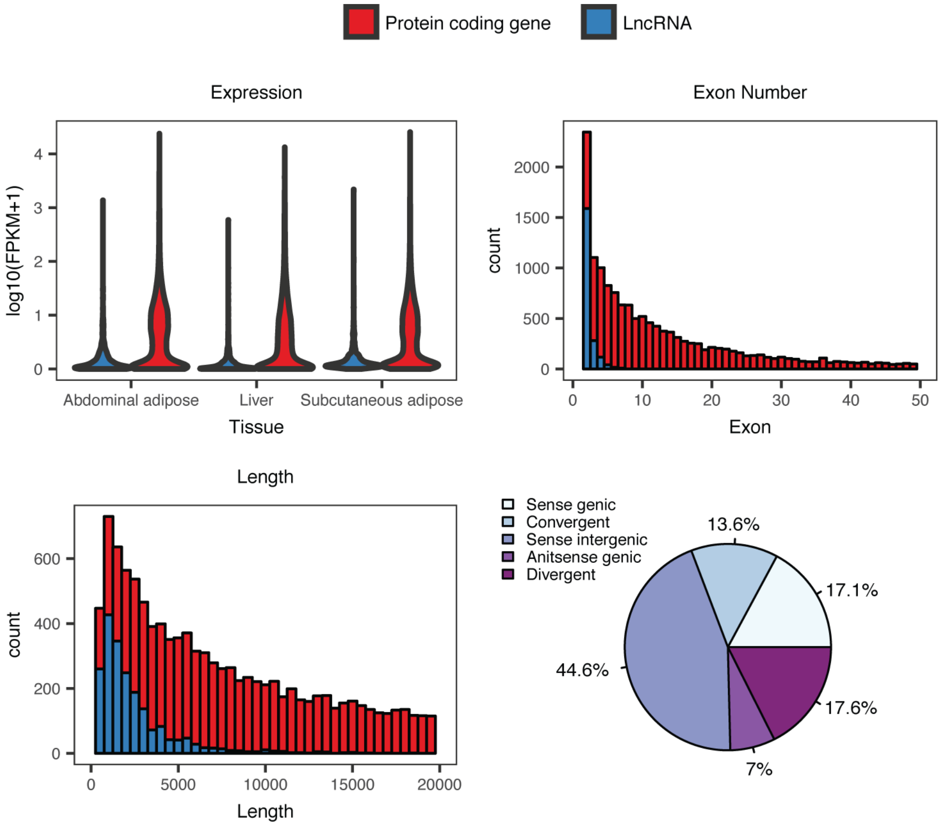

Figure S2. Comparison of characteristics of protein coding genes and lncRNAs and classification of lncRNAs.
