# Supplementary material for: Transcriptomic analysis between Normal and high-intake feeding geese provides insight into adipose deposition and susceptibility to fatty liver in migratory birds
Source: BMC Genomics. 2019 May 14;20:372. doi: 10.1186/s12864-019-5765-3 (PMC6518675; doi:10.1186/s12864-019-5765-3)
Supplement: Supplementary file 5 — Figure S5. Expression of mitochondria genes and important nuclear mitochondria-related genes. Asterisk mark indicates significantly differentially expressed between normal and high-dietary fed geese. (PDF 133 kb) [file 12864_2019_5765_MOESM5_ESM.pdf]

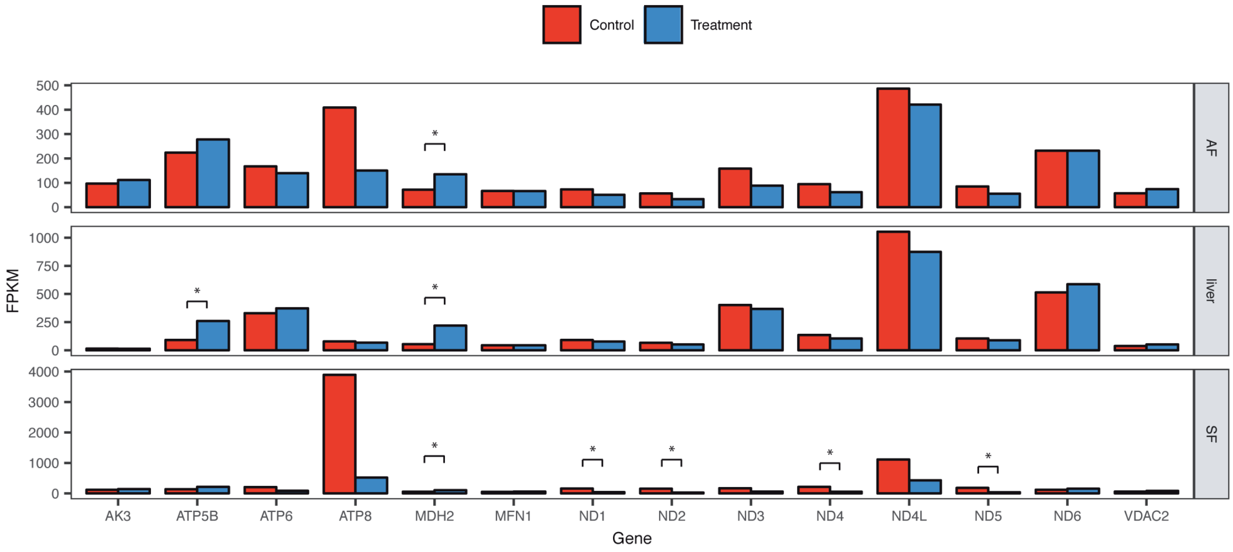

Figure S5. Expression of mitochondria genes and important nuclear mitochondria-related genes. Asterisk mark indicates significantly differentially expressed between normal and high-dietary fed geese.
