# Supplementary material for: Transcriptomic analysis between Normal and high-intake feeding geese provides insight into adipose deposition and susceptibility to fatty liver in migratory birds
Source: BMC Genomics. 2019 May 14;20:372. doi: 10.1186/s12864-019-5765-3 (PMC6518675; doi:10.1186/s12864-019-5765-3)
Supplement: Supplementary file 7 — Table S1. Paired t-test of body weights between normal and high dietary group during the fattening process. (PDF 63 kb) [file 12864_2019_5765_MOESM7_ESM.pdf]

| Day | t-statistics | *p*-value | Fold-change |
|---|---|---|---|
| Day 1 | -0.3176 | 0.758 | 1.105629 |
| Day 4 | -0.3708 | 0.7194 | 1.021425 |
| Day 7 | -2.1722 | 0.05791 | 1.105437 |
| Day 10 | -3.3754 | 0.008187 | 1.158945 |
| Day 13 | -4.8258 | 0.0009394 | 1.179954 |
| Day 16 | -5.0336 | 0.0007059 | 1.287549 |
| Day 19 | -4.9089 | 0.0008373 | 1.323378 |

**Table S1.** Paired t-test of body weights between normal and high dietary group during the fattening process.
