# Supplementary material for: Transcriptomic analysis between Normal and high-intake feeding geese provides insight into adipose deposition and susceptibility to fatty liver in migratory birds
Source: BMC Genomics. 2019 May 14;20:372. doi: 10.1186/s12864-019-5765-3 (PMC6518675; doi:10.1186/s12864-019-5765-3)
Supplement: Supplementary file 8 — Table S2. Paired t-test of relative tissue weight between normal and high dietary group. Relative weight = absolute weight/body weight. (PDF 70 kb) [file 12864_2019_5765_MOESM8_ESM.pdf]

Tissue	t-statistics	*p*-value	Fold-change
Liver	-4.6895	0.0011	3.3473
Abdominal adipose	-2.2602	0.0502	1.6541
Breast muscle	-0.1840	0.8581	1.0099
Leg muscle	-4.0206	0.0030	1.2622
Heart	0.0899	0.9303	0.9956
Duodenum	-2.3459	0.0436	1.2952
Gizzard	-0.1019	0.9210	1.0145
Glandular stomach	-3.2523	0.0099	1.3282
Spleen	-3.0499	0.0138	1.8256
Bursa of fabricius	4.6993	0.0011	0.5700

Table S2. Paired t-test of relative tissue weight between normal and high dietary group. Relative weight = absolute weight/body weight.
