# Supplementary material for: Transcriptomic analysis between Normal and high-intake feeding geese provides insight into adipose deposition and susceptibility to fatty liver in migratory birds
Source: BMC Genomics. 2019 May 14;20:372. doi: 10.1186/s12864-019-5765-3 (PMC6518675; doi:10.1186/s12864-019-5765-3)
Supplement: Supplementary file 9 — Table S3. Top 10 highly correlated mRNA-lncRNA pairs within 10Kb of each other. (PDF 77 kb) [file 12864_2019_5765_MOESM9_ESM.pdf]

lncRNA	mRNA	Coefficients	*p*-value	mRNA Description
XLOC_026991	B3GALT2	-0.994	4.21e-05	Galactosyltransferase
XLOC_031116	NCS1	0.986	3.01e-04	Ca, cAMP and lipid signaling
XLOC_002528	GJD2	0.983	3.45e-04	Development Slit-Robo signaling
XLOC_292472	FERMT2	0.977	8.07e-04	Phosphatidylinositol-3,4,5-triphosphate binding
XLOC_025867	DERL1	0.974	9.42e-04	Degradation in endoplasmic reticulum protein
XLOC_0292472	ERAL1	-0.971	1.22e-03	GTPase that localizes to the mitochondrion
XLOC_140330	FRMD4B	0.969	1.44e-03	Membrane response to insulin receptor signaling
XLOC_005028	HOXA10	0.960	2.32e-03	Transcription factor
XLOC_189739	RASSF8	0.952	3.34e-03	Maintaining adherens junction function in epithelial cells
XLOC_277626	ZFAT	0.928	7.60e-03	Transcription factor in regulation apoptosis and cell survival

Figure S3. Top 10 highly correlated mRNA-lncRNA pairs within 10Kb of each other.
